# Multi-Parametric Diffusion Tensor Imaging of The Optic Nerve for Detection of Dysthyroid Optic Neuropathy in Patients With Thyroid-Associated Ophthalmopathy

**DOI:** 10.3389/fendo.2022.851143

**Published:** 2022-05-03

**Authors:** Ping Liu, Ban Luo, Lin-han Zhai, Hong-Yu Wu, Qiu-Xia Wang, Gang Yuan, Gui-Hua Jiang, Lang Chen, Jing Zhang

**Affiliations:** ^1^ Department of Radiology, The Affiliated Tongji Hospital, Tongji Medical College, Huazhong University of Science and Technology, Wuhan, China; ^2^ Department of Medical Imaging, Guangdong Second Provincial General Hospital, Guangzhou, China; ^3^ Department of Ophthalmology, The Affiliated Tongji Hospital, Tongji Medical College, Huazhong University of Science and Technology, Wuhan, China; ^4^ Department of Radiology, Xiangyang Central Hospital, Affiliated Hospital of Hubei University of Arts and Science, Xiangyang, China; ^5^ Department of Endocrinology and Metabolism, The Affiliated Tongji Hospital, Tongji Medical College, Huazhong University of Science and Technology, Wuhan, China

**Keywords:** dysthyroid optic neuropathy, thyroid-associated ophthalmopathy, optic nerve, diffusion tensor imaging, magnetic resonance imaging

## Abstract

**Objective:**

To evaluate the microstructural changes of the orbital optic nerve in thyroid-associated ophthalmopathy (TAO) patients with or without dysthyroid optic neuropathy (DON) using diffusion tensor imaging (DTI) and investigate whether DTI can be used to detect DON.

**Materials and Methods:**

59 bilateral TAO patients with (n= 23) and without DON (non-DON, n= 36) who underwent pretreatment DTI were included and 118 orbits were analyzed. The clinical features of all patients were collected. DTI parameters, including mean, axial, and radial diffusivity (MD, AD, and RD, respectively) and fractional anisotropy (FA) of the intra-orbital optic nerve for each orbit were calculated and compared between the DON and non-DON groups. ROC curves were generated to evaluate the diagnostic performance of single or combined DTI parameters. Correlations between DTI parameters and ophthalmological characteristics were analyzed using correlation analysis.

**Results:**

Compared with non-DON, the DON group showed decreased FA and increased MD, RD, and AD (P < 0.01). In the differentiation of DON from non-DON, the MD was optimal individually, and the combination of the four parameters had the best diagnostic performance. There were significant correlations between the optic nerve’s four DTI metrics and the visual acuity and clinical active score (P < 0.05). In addition, optic nerve FA was significantly associated with the amplitude of visual evoked potentials (P = 0.022).

**Conclusions:**

DTI is a promising technique in assessing microstructural changes of optic nerve in patients with DON, and it facilitates differentiation of DON from non-DON eyes in patients with TAO.

## Introduction

Although accounting for only 3%–8% of thyroid-associated ophthalmopathy (TAO) (1), dysthyroid optic neuropathy (DON), is the most severe complication of TAO and can lead to blindness if treatment is untimely and ineffective. Visual loss from DON may be reversed if recognized and managed early and appropriately (2, 3); nevertheless, a majority of TAO patients may be unaware of the danger until the onset of pronounced visual loss in daily clinical practice. Thus, early recognition of DON is critical for decision-making and reducing the risk of permanent blindness.

The existing diagnostic criteria for DON are primarily based on clinical signs and symptoms, which are neither sensitive nor specific. Barrett et al. (4) suggested that imaging is particularly important for the evaluation of DON. MRI metrics, including the presence of fluid in the optic nerve sheath, muscle index, optic nerve diameter, optic nerve stretching, or apical crowding, have been previously used to detect DON (5–8). However, most of these methods were indirect manners and provided relatively little direct information about the pathological changes of the optic nerve in DON. Thus, conventional MRI is limited in differentiating DON from TAO, especially in patients with subclinical manifestations of visual dysfunction.

Diffusion tensor imaging (DTI) can directly detect subtle microstructural alterations of nerves (9) by quantifying the microscopic motion of water molecules within nerve fibers (10). DTI is sensitive to the underlying structural changes of nerves (11), it has been widely employed in optic neuropathies, including glaucoma (12, 13), multiple sclerosis (14), optic neuritis (15, 16), and amblyopia (17). Although there is a growing number of reports on the application of DTI in TAO (18–20), but they mainly focused on optic nerves in TAO patients without DON. We hypothesized that DTI could assess the underlying pathophysiology of optic nerve preceding its irreversible structural damage for DON while also being used as an auxiliary examination facilitating the diagnosis of DON.

Therefore, we aimed to investigate the microstructural changes in the optic nerves of TAO patients with and without DON using DTI and identify an imaging marker of the optic nerve to facilitate early diagnosis of DON. We also attempted to explore the relationship between DTI measurements and clinical characteristics.

## Materials And Methods

### Study Population

The study was approved by the Institutional Review Board of our hospital. TAO patients with or without DON were enrolled from the Ophthalmology or Endocrinology Department of our hospital. Written informed consents were obtained from all participants.

The diagnosis of TAO was based on the Bartley diagnostic criteria (21) and the European Group of Graves’ Orbitopathy consensus (22) as follows: a) if eyelid retraction occurred in association with objective evidence of thyroid dysfunction or abnormal regulation, exophthalmos, optic nerve dysfunction, or EOM involvement; and b) if eyelid retraction was absent, thyroid dysfunction was associated with exophthalmos, optic nerve dysfunction, or EOM involvement was adopted.

DON was diagnosed based on the following criteria with a and at least two indicators of b: a) a prerequisite history of TAO; and b) at least two of the following abnormal visual functions: unexplained decreased visual acuity (VA) <0.8; visual field defects (mean deviation in Humphrey perimetry <-10 dB); or relative afferent pupillary defects, impairment of color vision, abnormal pattern-visual evoked potentials (p-VEP, latency delay and amplitude reduction), as well as evident apical crowding in orbital CT.

The exclusion criteria were as follows: 1) concurrence of other neurological or ophthalmologic diseases that could influence visual function (multiple sclerosis, toxic, ischemic or inflammatory neuritis, cataract, glaucoma, macular diseases, uncorrected high astigmatism and myopia, congenital dyschromatopsia, diabetic retinopathy, etc.); 2) signs of severe corneal exposure (diffuse punctate keratopathy, ulcers, abscesses, leucomas, etc.); 3) steroid therapy, radiotherapy, or surgical decompression; 4) inadequate image quality; and 5) exclusion of DON for the TAO group.

### Clinical Features

Clinical features, including demographic characteristics, history of ^131^I treatment and smoking, duration of Graves’ disease, TAO and/or DON (the duration of eye signs was the time interval between the onset of abnormal symptoms and the time to consultation, mainly basing on the patient’s complaint), ophthalmologic assessment and current serologic levels of thyroid hormones and thyroid autoantibodies were collected. MRI examination was performed within three days for DON and one week for TAO before any initial treatment.

#### Ophthalmologic Assessment

Every patient underwent a complete ophthalmologic examination. Visual acuity, intraocular pressure (IOP), the degree of proptosis, mean deviation and pattern standard deviation of the visual field test, and average thickness of the peripapillary retinal nerve fiber layer were documented. VEP measurements (performed on pattern stimulation), including the amplitude and absolute latency of P100 (in ms) were collected. Additionally, the clinical activity score (CAS) was assessed based on the classical signs of inflammation. The CAS is comprised of seven items, including spontaneous retrobulbar pain, pain on attempted up- or down-gaze, redness of the eyelids, redness of the conjunctiva, swelling of the eyelids, inflammation of the caruncle and/or plica, and conjunctival edema.

#### Laboratory Measurements

Serum levels of free triiodothyronine (FT3), free thyroxine (FT4), thyroid-stimulating hormone (TSH), thyrotropin receptor antibody (TRAb), thyroglobulin autoantibody (TGAb), and thyroid peroxidase antibody (TPO-Ab) were measured *via* electrochemiluminescence immunoassay performed on a Cobas 6000 Analyzer (Roche Diagnostics, Mannheim, Germany). Reference ranges were defined as follows: FT3, 2.0‐4.4 pg/mL; FT4, 9.32‐17.09 ng/L; TSH, 0.27‐4.2 mIU/mL; TRAb, <1.58 IU/L; TGAb, <115 IU/mL; and TPO‐Ab, <34 IU/mL. Serum was collected on the day of visiting the clinician, and the MRI examination was performed within the following one week.

### MRI Technique: Image Acquisition and Image Processing

MR examinations were performed on a 3T scanner (Discovery 750, GE Healthcare, Milwaukee, WI, USA) with a 32Ch head coil. Participants were asked to remain still and keep their eyes closed during the scanning. Conventional MRI of the brain and orbit was performed to exclude brain and other optic visual pathway diseases.

For DTI, a single-shot echo-planar imaging sequence (TR/TE, 7800 ms/60 ms; flip angle, 20°; matrix, 512×512; field of view, 256×256; slice thickness, 2 mm; and slice gap, 0 mm) was applied with 64 non-collinear directions with b = 0 and 1000 s/mm^2^. The acquisition time was approximately 6 min 40 s, with 78 axial slices covering the whole brain.

**Figure 1:**
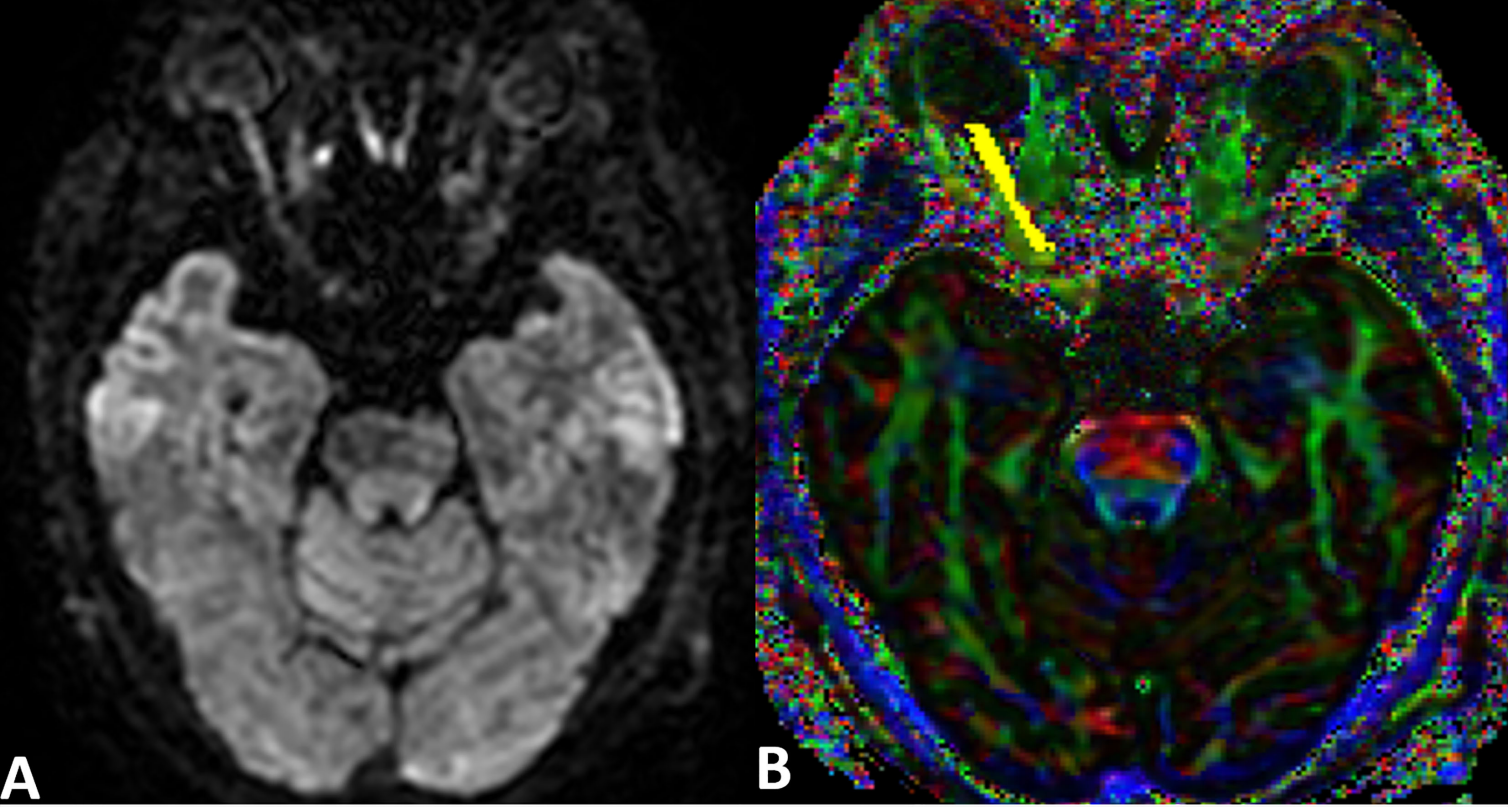
An example raw DWI image and DTI color map of the optic nerve. **(A)** A raw DWI image of the optic nerve. **(B)** A combined FA and directional color map of the optic nerve generated by MRI Studio. The region of interest is indicated in yellow. The color hue indicates direction as follows: red, left-right; green, anteroposterior; blue, superior-inferior.

DTI data processing was performed by two neuroradiologists, each with more than 5 years’ experience, who were blinded to the patient’s clinical status. All data processing was conducted using the open-source software MRI studio (www.mristudio.org) (23)as follows. The reconstructed volumetric optic nerve fiber is shown in **Figure 1**.

1) DTI computation: read the raw DTI data in a DICOM format. Additionally, the user visually inspected the individual images and discarded the corrupted images form possible motion-related phase errors.
2) Diffusion tensor calculation and visualization: the diffusion tensor-derived parameters including fractional anisotropy (FA), mean diffusivity (MD), axial diffusivity (AD) and radial diffusivity (RD) were calculated with multivariate linear fitting.
3) Fiber tracking: fibers were constructed using the Fiber Assignment by Continuous Tracking (FACT) approach, the fiber tracking started at the center of each voxel having a fractional anisotropy (FA) value greater than 0.2 and terminated at voxels where FA is lower than 0.3 or the tract turning angles between two eigenvectors to be connected by the tracking were above 70°.
4) Label interested nerve fiber bundle: the tract of each segment of the visual pathway were drawn on color-coded maps and real-time edited by operation tools including: AND (intersection), OR (union), and NOT (exclusion).Finally, each tract pathways of interest were selected, diffusion-related parameters (axial, radial and mean diffusivities) and an anisotropy index (fractional anisotropy) are studied for each part of visual pathway.

### Statistical Analysis

All statistical analyses were performed with the SPSS statistical software package (Version 25, IBM Corp., Armonk, NY, USA) and MedCalc (MedCalc Software, Mariakerke, Belgium). A significance level of P <0.05 was considered statistically significant, and all p values were based on two-tailed tests.

The two groups’ parameters were compared using Mann-Whitney U test for continuous variables and chi-square tests for categorical variables. Interclass correlation coefficients (ICC) for DTI parameters were calculated for all the enrolled patients to evaluate the two neuroradiologists’ measurement consistency. The Kolmogorov– Smirnov test was conducted to test the normality of DTI parameters. Independent-samples t-tests were used to compare the DTI measurements. Pearson’s correlation coefficient tests were used to analyze the association between DTI parameters and ophthalmologic variables. Receiver operating characteristic (ROC) curves of the DTI parameters (single or combined) were used to evaluate the diagnostic efficiency of discriminating patients with and without DON.

## Results

Fifty-nine bilateral TAO patients were included in this study. They were classified into the DON (n=23; 13 male, 10 female) and non-DON (n=36, 18 male, 18 female) groups. In total, 46 and 72 eyes from the DON and non-DON groups, respectively, were included in the analysis.

### Clinical Features

**Table 1** summarizes the patients’ clinical characteristics and demographics. Finally, 59 bilateral TAO patients with(n= 23) and without DON (non-DON, n= 36) who underwent pretreatment DTI were included and 118 orbits were analyzed. Compared to non-DON, DON patients had higher serologic TRAb levels and CAS, and decreased visual accuracy. However, the parameters of visual fields and visual evoked potential showed no difference between them. Which may due to the VEP are readily influenced by poor patient compliance and factors unrelated to the disease such as the opaque media, incorrect refraction and often render false positive results.

**Table 1:** Univariate analysis of the detailed clinical and demographic information of study population.

Variable	DON (N=23)	Non-DON (N=36)	*P* value
Age (year)	55.35 ± 8.89	52.14 ± 7.93	0.581
Gender (n, %)			0.598
Male	13 (56.5%)	18 (50%)	
Female	10 (43.5%)	18 (50%)	
Current thyroid status (n, %)			0.224
Euthyroid	6 (26.1%)	10 (27.8%))	
Hyperthyroid	11 (47.8%)	20 (55.6%)	
Hypothyroid	6 (26.1%)	6 (16.7%)	
Treatment			0.307
With I^131^	3 (13%)	8 (22.2%)	
Without I^131^	20 (87%)	28 (77.8%)	
Smoking			0.844
Yes	10 (43.5%)	14 (38.9%)	
No	13 (56.5%)	22 (61.1%)	
TSH (mU/L)	3.15 ± 8.23	4.02 ± 14.27	0.396
FT3 (pmol/L)	4.72 ± 4.04	3.45 ± 1.19	0.196
FT4 (pmol/L)	8.51 ± 7.19	7.58 ± 7.31	0.561
TPO	171.58 ± 201	105.36 ± 137.22	0.293
TGAb	481.69 ± 1126.29	193.96 ± 553.76	0.487
**TRAb**	**16.86 ± 11.16**	**10.56 ± 11.81**	**0.006**
Duration of GD (months)	4 (1–12)	11 (3-15)	0.059
Duration of TAO (months)	6 (2-10)	6 (3-12)	0.471
Duration of DON (months)	2 (0-6)	–	–
**CAS**	**2 (1-4)**	**2 (1-3)**	**0.002**
**VA**	**0.8 (0.5-1.0)**	**1 (0.8-1)**	**0.000**
Exophthalmos (mm)	19 (16.225-21)	18 (16-20)	0.09
IOP (mm Hg)	18 (17-20)	19 (17-21)	0.47
aRFNLT* (μm)	107.6 ± 22.4	103.34 ± 12.67	0.359
VEP*			
Latency (ms)	113.31 ± 15.1	109.72 ± 7.06	0.053
Amplitude (μV)	6 ± 3.9	7.88 ± 4.87	0.081
VF*			
VMD	-5.64 ± 7.3	-4.69 ± 1.9	0.163
PSD	5.44 ± 3.88	4.59 ± 1.69	0.078

Continuous variables are presented as the mean (± standard deviation) or as the median (interquartile range). Categorical variables are presented as the number (%) and counts. p < 0.05 was considered statistically significant.

TSH, thyroid stimulating hormone; FT3, free triiodothyronine; FT4, tetraiodothyronine; TGAb, thyroglobulin autoantibody; TPO-Ab, thyroid peroxidase antibody; TRAb, thyrotropin receptor antibody; CAS, clinical activity scores; VA, visual acuity; IOP: intraocular pressure; aRFNLT, average retinal nerve fiber layer thickness; VEP, visual evoked potential; VF, visual field;

VMD: mean deviation of visual field; PSD, pattern standard deviation.

^*^The current thyroid status was judged according to the serologic test within 2 weeks before MRI examination. The bold values indicate the matrix have statistical difference.

### Interobserver Agreement of DTI Parameters

The degree of inter-observer agreement between the two operators for measuring all DTI parameters was good to excellent (ICC > 0.822) (**Table 2**).

**Table 2:** Interobserver agreement of DTI parameters.

Parameters	DON	TAO
AD	0.913 (0.885,0.835)	0.876 (0.825, 0.912)
RD	0.888 (0.830,0.931)	0.815 (0.670, 0.855)
MD	0.833 (0.618, 0.963)	0.812 (0.713,0.857)
FA	0.829 (0.664, 0.965)	0.785 (0.705,0.857)

Data in parentheses are 95% confidence intervals.

ICC, intraclass correlation coefficient.

TAO, thyroid associated ophthalmopathy; DON, dysthyroid optic neuropathy.

AD, axial diffusivity; RD, radial diffusivity; MD, mean diffusivity; FA, fractional anisotropy.

### Group Differences in DTI Parameters

**Figure 2:**
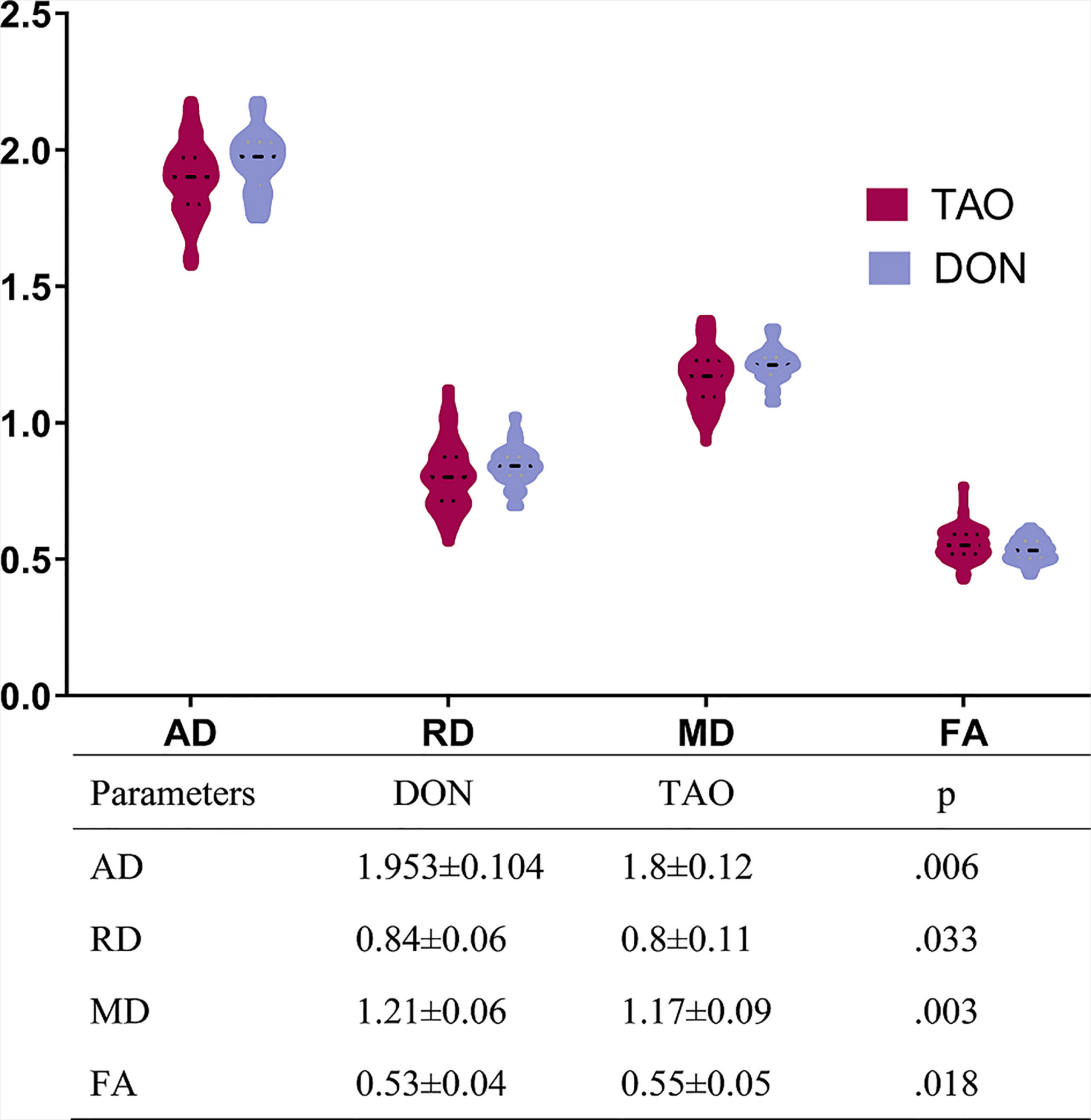
Comparisons of all DTI parameters of optic nerve between DON and non-DON. The above bar chart showing the comparison of DTI parameters between the DON and non-DON groups. There is a trend toward higher fluid AD, RD, and MD, but lower FA, in the DON group (p < 0.05, respectively). The table below displays the quantitative assessment of each DTI parameter. Units of MD, AD, and RD are x 10^-3^ mm^2^/s. A p-value < 0.05 was considered statistically significant. TAO, Thyroid-associated ophthalmopathy; DON, Dysthyroid optic neuropathy; AD, axial diffusivit; FA, fractional anisotropy; MD, mean diffusivity; RD, radial diffusivity.

**Figure 2** presents the DTI parameters of the optic nerve in all participants. The DON group had significantly higher AD, RD, and MD, and significantly lower FA values than those of the non-DON group (p= 0.006, 0.033, 0.003, 0.018, respectively).

### Diagnostic Performance of the DTI Parameters

**Figure 3:**
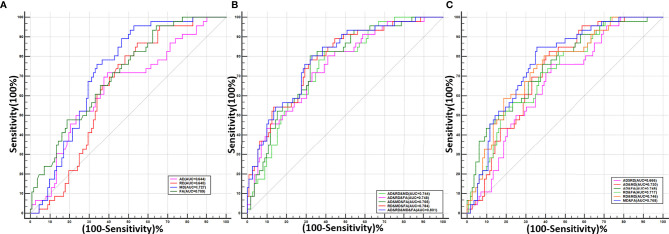
The receiver operating characteristic (ROC) curves for single or combined DTI parameters of the optic nerve to differentiate patients with and without dysthyroid optic neuropathy. **(A)** The ROC curves of the four single DTI parameters. **(B)** The ROC curves of the combination of any two DTI parameters. **(C)** The ROC curves of the combination of any three DTI parameters and all four parameters.

The diagnostic performances of DTI parameters are shown in **Table 3** and **Figure 3**. MD alone and a combination of the four DTI parameters generated the highest area under the ROC curve (AUC) (0.727 and 0.821, respectively), and the corresponding sensitivity and specificity of the combined performance were 80.43% and 66.96%, respectively.

**Table 3:** The receiver operating characteristics (ROC) analysis of single or combined DTI parameters of the optic nerve to differentiate patients with and without dysthyroid optic neuropathy (DON).

Parameters	AUC value	Cutoff value	Sensitivity (%)	Specificity (%)
AD	0.644 (0.564- 0.719)	>1.927	71.74	60.71
RD	0.640 (0.560-0.715)	>0.791	86.96	45.54
**MD**	**0.727 (0.650-0.794)**	>1.164	95.65	47.32
FA	0.709 (0.631-0.778)	≤0.574	95.65	35.71
AD+RD	0.666 (0.586-0.739)	≤0.711	76.09	57.14
AD+MD	0.720 (0.644-0.789)	≤0.719	82.61	58.04
AD+FA	0.748 (0.673-0.814)	≤0.728	76.09	61.61
RD+MD	0.746 (0.670-0.812)	≤0.716	80.43	60.71
RD+FA	0.717 (0.640-0.786)	≤0.713	71.74	62.5
**MD+FA**	**0.768 (0.695-0.832)**	≤0.738	84.78	64.29
AD+RD+MD	0.744 (0.668-0.81)	≤0.721	82.61	60.71
AD+RD+FA	0.748 (0.673-0.814)	≤0.736	80.43	58.93
AD+MD+FA	0.765 (0.691-0.828)	≤0.709	80.43	67.86
**RD+MD+FA**	**0.784 (0.712-0.846)**	≤0.727	78.26	67.86
**AD+RD+MD+FA**	**0.801 (0.739-0.842)**	≤0.729	80.43	66.96

AD, axial diffusivity; RD, radial diffusivity; MD, mean diffusivity; FA, fractional anisotropy.

AUC: area under curve.

Units of MD, AD, and RD are × 10^-3^ mm^2^/s.The bold values indicate the matrix have statistical difference.

### Correlation of DTI Parameters and Ocular Parameters

**Figure 4:**
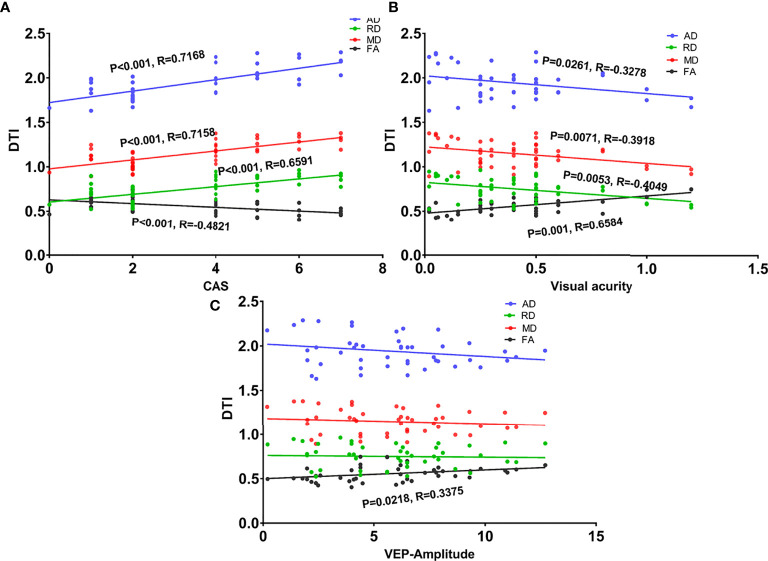
Scatter diagrams showing significant correlations between clinical indexes and DTI values of the intra-orbital optic nerve in the dysthyroid optic neuropathy group. **(A)** The correlation between the four DTI parameters and clinical activity scores (CAS). **(B)** The correlation between the four DTI parameters and visual acuity. **(C)** The correlation between the four DTI parameters and the amplitude of visual evoked potentials.

As shown in **Table 4** and **Figure 4**, the CAS and BCVA were significantly correlated with all DTI parameters. The VEP amplitude was also correlated with FA.

**Table 4:** Correlations of DTI Parameters and Ophthalmological Characteristics.

Parameters	AD		RD		MD		FA	
	r	P	r	P	r	P	r	P
CAS	0.717	<0.001*	0.659	<0.001*	0.716	<0.001*	-0.482	0.001*
VA	-0.328	0.026*	-0.405	0.005*	-0.392	0.007*	0.658	<0.001*
IOP	-0.189	0.208	-0.205	0.171	-0.208	0.165	0.182	0.227
Proptosis	0.111	0.463	0.002	0.989	0.049	0.749	-0.027	0.861
Latency	0.226	0.131	0.164	0.277	0.198	0.187	-0.265	0.075
Amplitude	-0.234	0.117	-0.045	0.766	-0.128	0.397	0.338	0.022*
VMD	-0.242	0.105	-0.104	0.493	-0.168	0.265	0.045	0.769
RFNLT	0.098	0.517	-0.028	0.851	0.024	0.874	-0.106	0.482

p < 0.05 was considered statistically significant.

TAO, thyroid associated ophthalmopathy; DON, dysthyroid optic neuropathy.

AD, axial diffusivity; RD, radial diffusivity; MD, mean diffusivity; FA, fractional anisotropy.

CAS, clinical activity score; VA, visual acuity; IOP, intraocular pressure.

VMD, mean deviation of visual field.

RFNL, thickness of peripapillary retinal nerve fiber layer.*Indicates the significant correlations between DTI parameters and ophthalmological characteristics in DON patients.

## Discussion

The detection of early visual function impairment of DON is often challenging due to overlapping symptoms with other conditions and the subjective-nature of existing auxiliary diagnostic tools (24). We proposed a multiple parametric DTI–based marker to assess the microstructural changes of the retrobulbar optic nerve and explore the early identification of DON. Our study’s main strength is that we used a method that directly assesses the optic nerve in DON. The results showed that: 1) the DTI indices of the optic nerve differed between DON and non-DON patients; DON patients showed increased AD, RD, and MD, and decreased FA; 2) DTI metrics of the optic nerve displayed a desirable diagnostic performance to differentiate DON from non-DON eyes; and 3) DTI parameters were correlated with some ophthalmological tests.

Clinically, a diagnosis of DON is suspected only when distinctive signs, such as decreased visual acuity, occur; however, these signs can overlap with other symptoms and lag behind the really onset of optic nerve damage. Although many other ophthalmologic-psychophysical or electrophysiological markers have been applied, these different methodologies tend to capture different aspects of the disease process and often render false-positive results. Especially in patients with bilateral DON, clinical features such as an afferent pupillary defect or color testing are less useful, whereas the widespread used optical coherence tomography is limited to retinal assessment.

In the current study, most of the general clinical measures, including the serum markers of thyroid function, disease duration, and most ophthalmic tests showed no distinct difference between the DON and non-DON groups. In consistent with the findings of Ponto et al. (25), we found that TRAb levels were indicative of DON, which suggests that autoimmune mechanisms may contribute to its onset and development. However, the results were less striking, and its diagnostic performance (AUC=0.703) was inferior to some single DTI values. Thus, TRAb alone cannot be considered as an independent and sufficient marker of DON. Further, it should be noted that the clinical presentation of DON can be heterogeneous. For example, optic disk swelling is not present in up to 50% of daily routine cases (26). Although visual acuity is often considered the most prominent feature of DON, it lags behind other symptoms and signs (27). Therefore, complementary markers should be taken into consideration during the diagnostic process of DON.

Optic nerve imaging using DTI directly characterizes the directional nature of water motion and reflects subclinical axonal and neuronal loss. It is more sensitive to pathologic changes of nerve fibers, even when no abnormalities are seen on conventional imaging modalities (28). This study showed that all four DTI metrics differed between the non-DON and DON groups. AD and RD were used to evaluate axonal integrity, and MD is associated with demyelination or glial cell impairment (29). FA quantifies the orientational coherence of diffusion and fiber integrity (30) and reflects fiber attenuation, axonal diameter, and myelination. Impairment due to loss of myelin and axons leads to decreased anisotropy and disruption of the nerves; changes in axonal membrane permeability lead to an increase in MD and a decrease in FA (31–33). In our study, we observed increased AD, RD, and MD, and decreased FA in patients with DON. Although the exact mechanisms of DON remain unclear, compression, stretch, ischemia, and even inflammatory optic neuritis have been proposed as possible causes (3), all of which can damage the optic nerves. A loss of large-type axons in the optic nerve’s proximal and orbital parts has been confirmed in a postmortem case report of a patient with DON (34). The underlying pathological changes of the optic nerves’ microstructure, reflected by our observed differences in DTI metrics, agreed with the immunohistochemical and ultrastructural findings of the specimens. This observation is identical to those of optic nerve studies regarding glaucoma (35), Leber’s hereditary optic neuropathy (36), and optic neuritis (37). Berna et al (18) also demonstrated increased MD and decreased FA in TAO patients, but their finding was limited to the midpoint of the optic nerve, and the comparison was performed between non- DON and healthy controls. In contrast, we focused on the distinction between TAO with and without DON, and calculated our DTI metrics based on the whole volumetric optic nerve bundle rather than on one or several regions of interest.

We found that MD was the single best diagnostic index (AUC, 0.727) among the four DTI parameters, which indicated that the eigenvalues might be more sensitive than FA in quantifying pathology (38). However, a combination of all four DTI parameters achieved the best performance (AUC, 0.801). It is noteworthy that DTI is a convenient tool that does not require complex setup or any active engagement from the patient, and multiple parameters can be obtained from a single scan. These markers can be used individually or combined to complement existing ophthalmological tests. Therefore, DTI of the optic nerve may help ophthalmologists objectively evaluate optic nerve abnormalities, especially in patients with subclinical manifestations of visual dysfunction, which may be used as an early surrogate to estimate the occurrence of DON and to make timely referrals. With verifying studies with larger sample sizes, DTI may one day be considered a definitive diagnostic method for DON.

Additionally, we found that DTI measures were correlated with visual acuity and CAS, suggesting that optic neuropathy progression is related to visual acuity and CAS in DON. The CAS represents TAO activity, and a higher score indicates that the disease is at an inflammatory stage. Along with the increased AD, RD, and MD, which reflect enhanced water diffusion, this suggests that inflammation-mediated optic neuritis may play a role in developing neuropathy during TAO. This may partially explain why a large dose of methylprednisolone is recommended as the first-line therapy for DON. In the study by Naismith et al (39), increased RD was associated with decreased visual acuity, this correlation suggests that DTI parameters can reflect the severity of visual function. P-VEP quantifies the electrical manifestation of the brain’s response to external stimulation (40). The association between FA and the amplitude of VEP indicates that the electrical signal is associated with fiber integrity. Similar correlations have been reported by Wang et al. (38) regarding DTI measure and subacute anterior ischemic optic neuropathy.

Our study has several limitations. First, the population size was relatively small, which may affect the statistical power; thus, it is difficult to conclude the accuracy of DTI to predict the progression of TAO patients with and without DON. Second, this study applied multi-parametric DTI; future studies using advanced post-processing such as non-Gaussian (diffusion kurtosis) modeling or K-means clustering algorithms may provide additional insights. Third, this was a single-center study, and results from large, multi-center are need to support the role of DTI in the clinical diagnosis of DON. Last, lack of “gold standard” of the pathologic changes of optic nerve, such as histological or immunohistological analyses, making the results is a little less convincing.

In summary, DTI measurements within the intra-orbital optic nerve can detect microstructural changes of optic nerves and effectively distinguished TAO with DON from non- DON. With additional data from future larger-scale longitudinal validation studies, DTI measures could prove useful in combination with other imaging and non-imaging biomarkers in identifying patients with TAO at greatest risk for DON and monitoring the effects of treatments administered during the preclinical stage. Moreover, the correlation between DTI parameters of the optic nerve and ophthalmic tests suggests that these parameters play a role as disease indicators. This has significant implications for the study of patients in the early stages of neuropathy, permitting an earlier diagnosis and initiation of medical treatment and enabling clinicians to better manage DON.

## Data Availability Statement

The raw data supporting the conclusions of this article will be made available by the authors, without undue reservation.

## Ethics Statement

The studies involving human participants were reviewed and approved by the institutional review board of Tongji hospital. The patients/participants provided their written informed consent to participate in this study.

## Author Contributions

JZ and BL: was involved in the conception and design of the study. PL, BL, H-yW, L-hZ, LC, QX-W, and GY: were involved in the data collection. JZ and PL: were involved in the analysis and interpretation of the data. PL: wrote the first draft of the manuscript. JZ, BL, and Gh-J: revising the article critically for important intellectual content. All authors read and approved the final paper.

## Funding

This work was supported by grants from the Youth Program of the National Natural Science Foundation of China (No. 82102004), National Natural Science Foundation of China (No. 81771793), 3D Printing Scientific Research Project Foundation of Guangdong Second Provincial General Hospital (3D-A2021013), Medical Science and Technology Research Foundation of Guangdong Province (A2021220) and the Young Science Foundation of Guangdong Second Provincial General H ospital (2019-QNJJ-01).

## Conflict of Interest

The authors declare that the research was conducted in the absence of any commercial or financial relationships that could be construed as a potential conflict of interest.

## Publisher’s Note

All claims expressed in this article are solely those of the authors and do not necessarily represent those of their affiliated organizations, or those of the publisher, the editors and the reviewers. Any product that may be evaluated in this article, or claim that may be made by its manufacturer, is not guaranteed or endorsed by the publisher.
